# A case of bronchiolar adenoma/ciliated muconodular papillary tumor in the pulmonary center with high FDG accumulation on PET

**DOI:** 10.1186/s44215-023-00052-3

**Published:** 2023-07-17

**Authors:** Naoki Yamashita, Tomomi Hirata, Noriko Motoi, Toshihiko Iizuka, Satoru Kakuta, Nobuhiro Yamazaki, Yuki Nakajima, Hiroyasu Kinoshita, Hirohiko Akiyama

**Affiliations:** 1 Department of Thoracic Surgery, Saitama Cancer Center, 780, Komuro, Ina, Kita-Adachi-gun, Saitama, 362-0806 Japan; 2 Department of Pathology, Saitama Cancer Center, Saitama, Japan

**Keywords:** Bronchiolar adenoma/ciliated muconodular papillary tumor, BA/CMPT, PET-CT, Lung tumor

## Abstract

**Background:**

Bronchiolar adenoma/ciliated muconodular papillary tumor (BA/CMPT) is listed in the World Health Organization (WHO) Classification 5th edition as a rare benign tumor with papillary growth of ciliary, goblet, and basal cells.

**Case presentation:**

The patient was a 67-year-old female in whom a nodular shadow of 20 mm in diameter in the right lower lobe S10 center was found in chest computed tomography (CT) for examination of dorsal pain. Positron emission tomography/computed tomography (PET-CT) showed the accumulation of ^18^F-fluorodeoxyglucose (FDG) with a standardized uptake value (SUV)_max_ of 13.0. Primary lung cancer was suspected, and surgery was scheduled as a therapeutic strategy. Thoracoscopic resection of the right lower lobe was performed, and possible BA/CMPT or adenocarcinoma was suggested in the differential diagnosis by the intra-perioperative rapid pathologic diagnosis. The final diagnosis was BA/CMPT. Histological findings indicated that the cause of the high FDG-PET scan might be due to many inflammatory cell infiltration in the tumor.

**Conclusions:**

We report a resected case of BA/CMPT with exceptionally high FDG accumulation in PET.

## Introduction

Bronchiolar adenoma/ciliated muconodular papillary tumor (BA/CMPT) is a rare benign tumor included in the WHO Classification 5th edition. The tumor often develops in the peripheral lung and has a low standardized uptake value (SUV)_max_ on ^18^F-fluorodeoxyglucose (FDG) positron emission tomography/computed tomography (PET/CT), which makes differentiation from primary lung cancer difficult. Here, we report our experience in a case of BA/CMPT that developed in the pulmonary center with high FDG accumulation in PET and was also difficult to differentiate from lung cancer.

## Case presentation

The patient was a 67-year-old non-smoker female who had visited a nearby hospital for a chief complaint of dorsal pain. A suspected node in the lower lobe of the right lung was detected on chest CT, and thus, the patient was introduced to our hospital. She had comorbidities of hypertension, diabetes, and cataract and a history of cerebral hemorrhage. At the first examination, physical findings were height 153.1 cm, weight 66.8 kg, and no abnormal findings. Biochemical tests and tumor markers were normal. Respiratory function was *VC* 2.96 L (*%VC* 117.0%), *FVC* 2.64 L (*%FVC* 114.8%), *FEV1* 1.79 L (*%FEV1* 67.8%), *DLCO* 14.36 (*%DLCO* 79.2%).

**Fig. 1:**
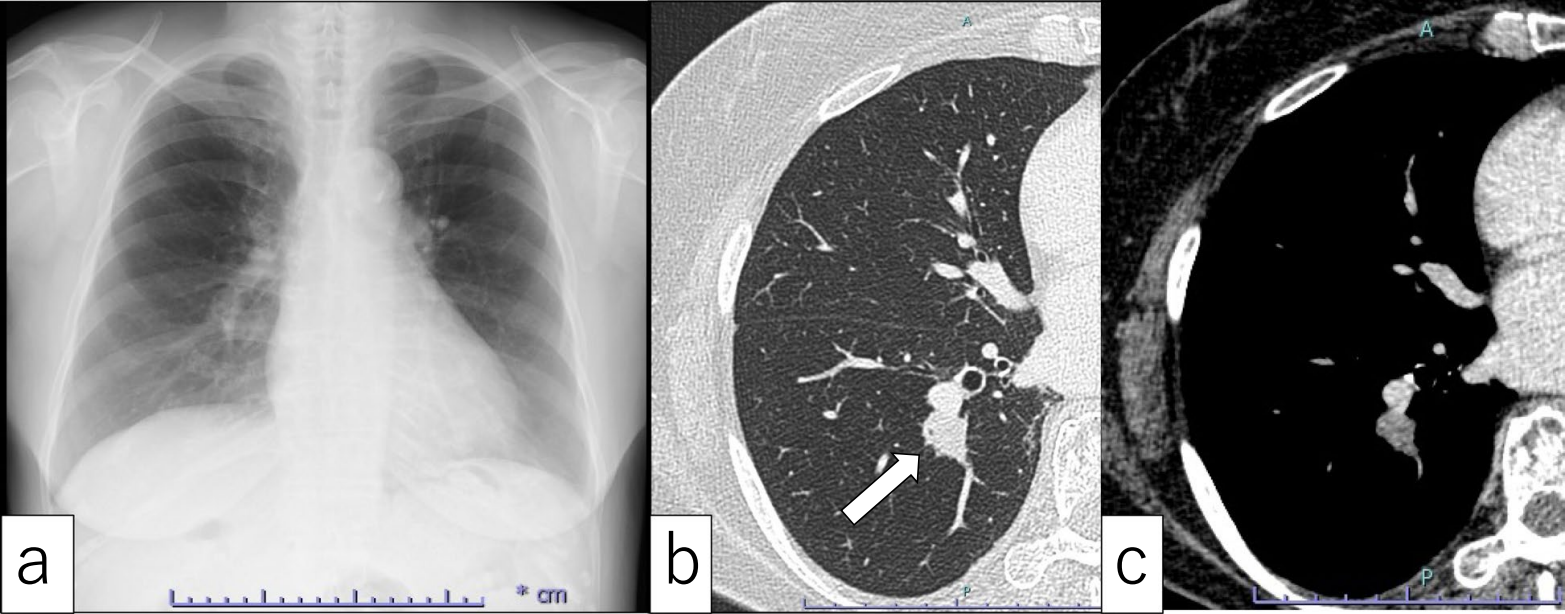
**a** Chest radiograph showing no obvious abnormal shadows. **b** Axial CT showed a nodule of 20 mm in diameter on the central side of S10 in the right lower lobe of the lung (arrow). **c** Axial contrast CT of the mediastinal window showed a relatively low density nodule with weakly heterogenous concentration

**Fig. 2:**
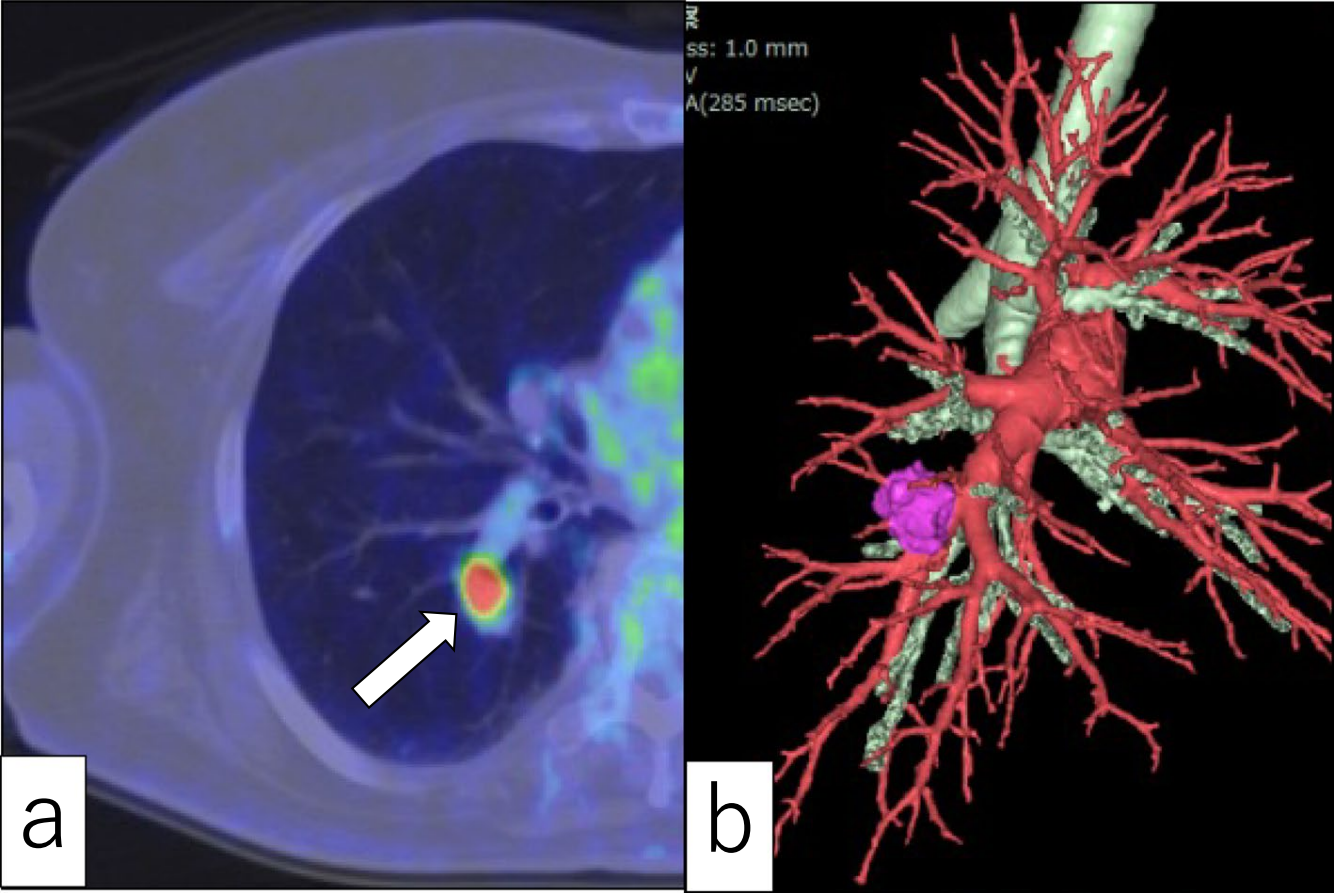
**a** Axial FDG-PET-CT fusion showed a nodule (arrow) with high FDG uptake (SUV_max_ 13.01) in the right lower lobe of the lung. **b** 3D-CT of the right pulmonary artery and bronchus confirmed that the tumor was located in the pulmonary center. Tumor, pink; bronchus, white; arteries, red

Chest X-ray showed no abnormal findings in pulmonary fields, and it was difficult to identify a node (Fig. 1a). However, chest CT showed a nodular shadow with a solid region of 20 mm in diameter in the right lower lobe S10 center (Fig. 1b). Axial contrast CT of the mediastinal window showed a relatively low-density nodule with weakly heterogenous concentration (Fig. 1c). Chest CT conducted about 1 month later showed no change in the tumor size and no clear enlargement of hilar and mediastinal lymph nodes. On PET/CT, the S10 lesion in the right lower lobe showed abnormal accumulation of FDG with a SUV_max_ of 13.0 (Fig. 2a). In bronchoscopy performed after PET, 5 samples from biopsy of right B10a were obtained for brush cytology and lavage cytology. The diagnosis was class 2 in this cytology, but no definite diagnosis was made because lung tissue was suggested in tissue diagnosis.

Primary lung cancer could not be excluded based on these findings. Thus, the case was clinically diagnosed as primary right lung cancer (cT1cN0M0, stage 1A3), and surgery was scheduled for diagnostic therapy. Four-port thoracoscopic surgery was performed. Since the tumor was present in the pulmonary center (Fig. 2b), the right lower lobe was initially resected to allow for perioperative rapid pathologic diagnosis. To include BA/CMPT and adenocarcinoma in the differential diagnosis, mediastinal lymphadenectomy was additionally performed before completion of surgery.

**Fig. 3:**
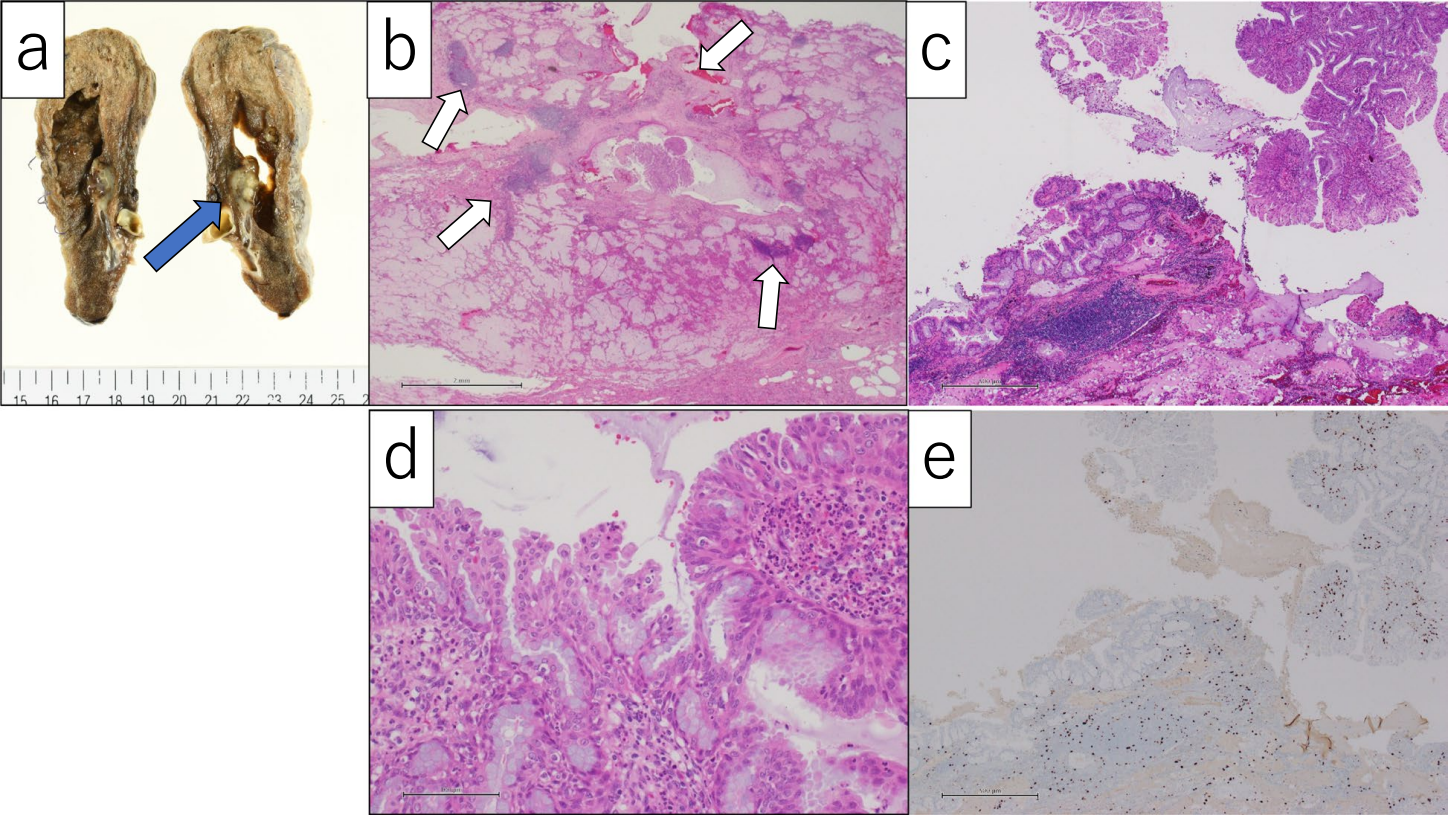
**a** Macroscopic cut surface of the resected tumor shows a mucinous nodular lesion, measuring 20 mm in diameter in the right lower lobe of the lung (blue arrow). **b** Low-power view of the tumor shows abundant mucin in the alveolar spaces and papillary growth pattern, involving a bronchiole. Moderate lymphocytic infiltrate (white arrows) was observed within the tumor, often forming lymph follicles (HE stain, scale bar: 2 mm). **c** Representative image of inflammatory infiltrates in the tumor. There are lymphocyte-infiltrated (left part) and neutrophilic-infiltrated (right upper part) areas. Many macrophages are also observed in the alveolar spaces with mucin (lower part) (HE stain, scale bar 500 μm). **d** High-power view of the tumor reveal ciliated columnar cells, goblet cells, and basal cells with no atypia. Note many neutrophilic infiltrates in the epithelium and stroma (HE stain, scale bar: 100 μm). **e** Immunohistochemical staining of Ki-67 (MIB1) on the serial section of **c**. Ki-67-immunoreactivity is found on the infiltrated lymphocytes (left) and basal cells in the inflamed area (right upper). Tumoral columnar and goblet cells are mostly negative (IHC, DAB visualization, scale bar: 500 μm)

Histopathologically, the tumor was diagnosed as BA/CMPT which showed mucinous nodular mass with papillary or lepidic growth of bronchiolar epithelium, including mucous, ciliated, and basal cells (Fig. 3b–d). No aggressive epithelial growth or cellular atypia was seen. Many inflammatory cells infiltrated within the tumor, including lymphocytes, neutrophils, and macrophages, suggesting subacute inflammation (Fig. 3c–d). Inflammatory cells and tumoral basal cells in the inflamed area showed immunoreactivity for Ki-67, indicating higher mitotic activity than tumoral ciliated or mucinous cells (Fig. 3e). The thoracostomy tube was removed on postoperative day (POD) 2, and the patient was discharged from hospital on POD 8. The patient is currently alive with no redevelopment in 1 year and 3 months since surgery.

## Discussion

BA/CMPT develops in the peripheral lung with mucus production and was first reported as a rare tumor, CMPT, which shows alveolus lepidic and papillary growth of biphasic ciliary, goblet, and basal cells. In 2021, the tumor was listed as bronchiolar adenoma including a nonclassical type with less papillary growth in the WHO Classification 5th edition [1]. The epidemiological characteristics of BA/CMPT include no sex difference and development at age 60 to 80 years (mean: 70.1 years) [2, 3]. Most BA/CMPT cases have been reported from Japan and other Asian countries and may involve genetic mutations of BRAF, EGFR, KRAS, HRAS, and ALK; thus, the tumor is viewed as an oncological disease [4–6].

CT findings for BA/CMPT show several types of frosted glass-like or solid nodes, with differences in sizes depending on the amount of mucus components in pathological findings. In a study in 16 patients with BA/CMPT, Onishi et al. found a mean nodular size of 9.1 mm, with most tumors present in the lower lobe (13/16, 81.3%) and > 50% of the tumors located close to the visceral pleura [3]. Previous findings have suggested that BA/CMPT of ≥ 15 mm in diameter is rare [7]. In FDG/PET-CT in fifteen patients with BA/CMPT, the SUV_max_ in 14 cases ranged from 0.57 to 1.35, with no significant increase in accumulation in most nodes. This suggests involvement of a large amount of mucus and less lymphoid infiltration [8] and makes it difficult to differentiate BA/CMPT from pulmonary adenocarcinoma in the early phase based on SUV_max_ alone. Based on pathological findings in our case, the abnormally high SUV_max_ may be due to concomitant subacute inflammation, as there was many lymphocytic and neutrophilic infiltrate within the tumor. However, primary lung cancer was strongly suspected based on preoperative examinations, as well as the large tumor size and high SUV_max_, and thus, lobe resection was chosen to ensure inclusion of the tumor margin.

We used SUV_max_ of 2.5, which is useful for diagnosis of malignant or benign tumors, as a cutoff for BA/CMPT cases with an abnormally high SUV_max_ [8]. A search using “BA/CMPT, PET-CT” on database (PubMed and Ichushi Web) identified 6 cases with SUV_max_ ≥ 2.5 (Table 1) [9–13]. The tumor sizes in these cases ranged from 7 to 20 mm (mean: 14.6 mm), and all tumors were in the lower lobe. Excluding one case and our case, all were peripheral lesions. PET-CT showed FDG accumulation with SUVmax 2.7–13.0 (mean: 5.3). Most patients underwent partial resection, excluding our case. These findings suggest that most patients with high FDG accumulation have a larger tumor compared with other BA/CMPT cases, as mentioned above. Perioperative pathological diagnosis was reported in one case, in which it was difficult to differentiate the lesion from a malignant tumor, as in our case, and resection of the lung lobe was conducted. The tumor size was larger, and FDG accumulation was higher in our case than in all six previously reported cases.

**Table 1:** BA/CMPT cases with SUV_max_ ≥ 2.7 on FDG/PET-CT

Author	Age	Sex	Size (mm)	Location		CT finding	SUV_max_	Intraoperative rapid diagnosis	Operation	Outcome (months)
Onishi [8]	65	M	14	RLL	N/a	N/a	3.67	N/a	N/a	N/a
Wang [9]	64	F	12	Rt-S10	Peripheral	Solid nodule	4.36	N/a	Wedge resection	N/a
Shirsat [10]	71	F	20	RLL	Peripheral	Solid nodule	5.4	N/a	Wedge resection	N/a
Patané [11]	68	F	20	Rt-S8	Peripheral	Solid nodule	2.7	Malignancy	Lobectomy	N/a
Krishnamurthy [12]	70	F	9	RLL	Peripheral	Solid nodule	5.4	N/a	Wedge resection	N/a
	74	F	7	LLL	Peripheral	Solid nodule	2.7	N/a	Wedge resection	N/a
Our case	67	F	20	Rt-S10	Central	Solid nodule	13.01	BA/CMPT or adenocarcinoma	Lobectomy	14

*BA/CMPT* Bronchiolar adenoma/ciliated muconodular papillary tumor, *RLL* Right lower lobe, *Rt* Right, *LLL* Left lower lobe, *n/a* Not available

Pathological diagnosis of BA/CMPT on frozen section has difficulties of differentiation from mucinous adenocarcinoma and mucous cell metaplasia. BA/CMPT has fewer atypical mucous cells, the presence of ciliary cells, and biphasic bronchoepithelial cells and basal cells, as important characteristics [14]. Shirsat et al. found that only 16.7% (3/18) cases of CMPT could be diagnosed using operative rapid pathologic diagnosis alone. In most cases, CMPT was underrecognized and misdiagnosed as primary lung adenocarcinoma, and this may result in unnecessary treatment [15]. Frozen diagnosis has limitation of the quantity and quality of sample; thus, surgeons should know the limitation of diagnostic accuracy. For the accurate diagnosis, surgeons and pathologists should share the patient’s information, including radiological findings and clinical course. If a sufficient surgical margin can be ensured in surgery for BA/CMPT, radical cure is possible, and no redevelopment and metastasis have been reported to date. Limited surgery should be selected, and segmentectomy and lung lobectomy are not needed, other than for securing the stump. As mentioned above, rapid diagnosis is difficult, and care should be taken to avoid an erroneous diagnosis of mucinous adenocarcinoma.

Since our case had a large tumor and an abnormally high SUV_max_, primary lung cancer was strongly suspected. Furthermore, the tumor developed in the pulmonary center, and thus, lobectomy was appropriate. A search for “BA/CMPT, pulmonary center” in PubMed/Ichushi Web identified four cases (Table 2) [16–19]. The tumor size and FDG accumulation in our case were larger than those in the four previous cases. Lobe resection was selected for the pulmonary center lesions in all five cases. This reflects the difficulty of ensuring the margin using partial or segmental resection for lesions in this location, and thus, lobectomy is performed. However, care is required to differentiate cases with high FDG accumulation from primary lung cancer. Thus, it is important to diagnose and treat patients with a pulmonary tumor with inclusion of BA/CMPT in differential diagnosis before surgery.

**Table 2:** BA/CMPT cases occurring in the pulmonary center

Author	Age	Sex	Size (mm)	Location		CT finding	SUV_max_	Intraoperative rapid diagnosis	Operation	Recurrence	Outcome (months)
Ishikawa [15]	50	F	15	RUL	Central	Solid nodule	N/a	BAC	Lobectomy	-	120
Hata [16]	76	F	7	LLL	Central	Solid nodule	No uptake	Neoplasia	Lobectomy	-	23
Kon [17]	66	M	14	RLL	Central	Solid nodule	N/a	Lung cancer	Lobectomy	-	14
Ono [18]	75	F	6	RLL	Central	Solid nodule	1.9	N/a	Lobectomy	-	5
Our case	67	F	20	RLL	Central	Solid nodule	13.01	BA/CMPT or adenocarcinoma	Lobectomy	-	14

*BA/CMPT* Bronchiolar adenoma/ciliated muconodular papillary tumor, *RUL* Right upper lobe, *RLL* Right lower lobe, *LLL* Left lower lobe, *n/a* Not available

## Data Availability

All data are included in the report.
